# Ammonium chloride reduces excitatory synaptic transmission onto CA1 pyramidal neurons of mouse organotypic slice cultures

**DOI:** 10.3389/fncel.2024.1410275

**Published:** 2024-10-01

**Authors:** Dimitrios Kleidonas, Louis Hilfiger, Maximilian Lenz, Dieter Häussinger, Andreas Vlachos

**Affiliations:** ^1^Department of Neuroanatomy, Institute of Anatomy and Cell Biology, Faculty of Medicine, University of Freiburg, Freiburg, Germany; ^2^Heinrich Heine University Düsseldorf, Düsseldorf, Germany; ^3^Center BrainLinks-BrainTools, University of Freiburg, Freiburg, Germany; ^4^Center for Basics in NeuroModulation (NeuroModulBasics), Faculty of Medicine, University of Freiburg, Freiburg, Germany

**Keywords:** ammonium chloride, excitatory neurotransmission, astrocytes, glutamine synthetase, CA1

## Abstract

Acute liver dysfunction commonly leads to rapid increases in ammonia concentrations in both the serum and the cerebrospinal fluid. These elevations primarily affect brain astrocytes, causing modifications in their structure and function. However, its impact on neurons is not yet fully understood. In this study, we investigated the impact of elevated ammonium chloride levels (NH_4_Cl, 5 mM) on synaptic transmission onto CA1 pyramidal neurons in mouse organotypic entorhino-hippocampal tissue cultures. We found that acute exposure to NH_4_Cl reversibly reduced excitatory synaptic transmission and affected CA3-CA1 synapses. Notably, NH_4_Cl modified astrocytic, but not CA1 pyramidal neuron, passive intrinsic properties. To further explore the role of astrocytes in NH_4_Cl-induced attenuation of synaptic transmission, we used methionine sulfoximine to target glutamine synthetase, a key astrocytic enzyme for ammonia clearance in the central nervous system. Inhibition of glutamine synthetase effectively prevented the downregulation of excitatory synaptic activity, underscoring the significant role of astrocytes in adjusting excitatory synapses during acute ammonia elevation.

## Introduction

Hepatic encephalopathy (HE) is a neurological disorder caused by acute liver failure, chronic liver disease (Haussinger et al., 2022; Wijdicks, 2016), and liver-independent portosystemic shunts (Hawkes et al., 2001; Vilstrup et al., 2014). The disorder manifests a spectrum of neuro-psychiatric symptoms ranging from subtle changes detectable only through specialized testing to severe cognitive and motor impairments, and in extreme cases, death (Rose et al., 2020; Vidal-Cevallos et al., 2022; Vilstrup et al., 2014). A prominent feature of HE is hyperammonemia, defined as abnormally high ammonia levels in the blood (Ong et al., 2003). Ammonia can cross the blood–brain interface (Jayakumar and Norenberg, 2018; Lockwood et al., 1979; Vidal-Cevallos et al., 2022) leading to cognitive and motor dysfunctions (Butz et al., 2010; Felipo, 2013; Garcia-Garcia et al., 2018), disorientation (Weissenborn, 1998), asterixis, which is characterized by an inability to sustain posture and involuntary movements (Zackria and John, 2023), and coma (Bessman and Bessman, 1955; Wijdicks, 2016).

Animal models (DeMorrow et al., 2021) have played a crucial role in elucidating the neurological consequences of increased ammonia levels in HE (Lima et al., 2019). Neuroinflammation is now recognized as a significant factor in both acute and chronic HE, as evidenced by research using various animal models, including the ammonium-containing diet model (Rodrigo et al., 2010), the bile duct ligation model (Claeys et al., 2022; Rodrigo et al., 2010), and models of acute liver failure (Jiang et al., 2009a; Jiang et al., 2009b; Zemtsova et al., 2011). Furthermore, changes in astrocytic structure, such as swelling (Jayakumar et al., 2009; Jayakumar et al., 2014b; Rama Rao et al., 2010), altered function (Drews et al., 2020; Zielinska et al., 2022), and senescence (Gorg et al., 2015), have been consistently observed in response to hyperammonemia (Ali and Nagalli, 2023).

Astrocytes, integral to neuronal communication (Adermark et al., 2022; Akther and Hirase, 2022; Dallerac et al., 2013; Farhy-Tselnicker et al., 2021; Santello et al., 2019), are believed to be involved in the synaptic modifications induced by ammonia (Jayakumar et al., 2014a; Rangroo Thrane et al., 2013). Several studies have identified ammonia-induced neuronal changes (Kelly and Church, 2005; Sancho-Alonso et al., 2022b), which have been linked to impaired astrocytic function in clearing glutamate and potassium (Rangroo Thrane et al., 2013). Astrocytes express glutamine synthetase, which converts ammonia and glutamate into glutamine, aiding in ammonia detoxification and glutamate clearance (Rose et al., 2013; Suarez et al., 2002). Additionally, astrocytic excitatory amino acid transporters (EAATs) are critical for glutamate re-uptake at synapses and neuronal functionality (Limon et al., 2021; Todd and Hardingham, 2020). Dysfunctions in these astrocytic EAATs have been associated with HE pathology in animal models (Knecht et al., 1997; Rose, 2006), with high ammonia levels leading to cognitive impairments, fear memory deficits, and seizures (Balzano et al., 2020; Cabrera-Pastor et al., 2016; Llansola et al., 2015; Qvartskhava et al., 2015; Rangroo Thrane et al., 2013; Rodrigo et al., 2010). Despite these insights, the exact cellular mechanisms by which ammonia induces changes in synaptic activity and transmission are still not fully understood.

This study investigated the acute effects of ammonium chloride (NH_4_Cl) on excitatory synaptic activity in 3-week-old entorhino-hippocampal tissue cultures of mice. These cultures maintain a cytoarchitecture and fiber organization that resemble *in vivo* conditions, allowing for the examination of neurons and glial cells without the need for interventions such as anesthesia, brain extraction, and slice preparation immediately before experimental assessment. We employed single-cell and paired whole-cell CA3-CA1 recordings to assess the acute effects of NH_4_Cl on synaptic transmission and passive membrane properties of CA1 neurons and astrocytes. Notably, the effects of NH_4_Cl on synaptic plasticity induction have been studied before at Schaffer collateral-CA1 synapses (c.f., Fan and Szerb, 1993). Our results revealed that short exposure to 5 mM NH_4_Cl significantly decreased spontaneous excitatory synaptic activity. This reduction in synaptic transmission coincided with astrocyte depolarization. Importantly, when we inhibited glutamine synthetase, the NH_4_Cl-induced suppression of excitatory transmission was counteracted.

## Materials and methods

### Ethics statement

Mice were maintained in a 12 h light/dark cycle with food and water available *ad libitum*. Every effort was made to minimize distress and pain of animals. All experimental procedures were performed according to German animal welfare legislation and approved by the appropriate animal welfare committee and the animal welfare officer of Freiburg University (AZ X-17/07 K).

### Animals

Wild type *C57BL/6J* mice of either sex were used in this study.

### Preparation of tissue cultures

Organotypic entorhino-hippocampal tissue cultures were prepared at postnatal day 4–5 from *C57BL/6J* mice of either sex as previously described (Del Turco and Deller, 2007). Briefly, mice were rapidly decapitated and their brains were quickly extracted and transferred to a Vibratome VT1200S (Leica) for 300 μm slicing. The tissue cultures were transferred for cultivation onto porous (0.4 μm pore size, hydrophilic PTFE) cell culture inserts with 30 mm diameter (Millipore, Cat# PICM0RG50). The culturing medium consisted of 50% (v/v) minimum essential medium (MEM; Gibco, Cat# 21575–022), 25% (v/v) basal medium eagle (BME; Gibco, Cat# 41010–026), 25% (v/v) heat-inactivated normal horse serum (NHS; Gibco, Cat# 26050–088), 2 mM GlutaMAX (Gibco, Cat# 35050–038), 0.65% (w/v) glucose (Sigma, Cat# G8769), 25 mM HEPES buffer solution (Gibco, Cat# 15630–056), 0.1 mg/mL streptomycin with 100 U/mL penicillin (Sigma, Cat# P0781) and 0.15% (w/v) bicarbonate (Gibco, Cat# 25080–060). The pH of the culturing medium was adjusted to 7.30 and tissue cultures were incubated for at least 18 days at 35°C in a humidified atmosphere with 5% CO_2_. The culturing medium was replaced thrice a week.

### Pharmacology

Organotypic entorhino-hippocampal tissue cultures (≥18 days *in vitro*) were acutely exposed to NH_4_Cl (5 mM; Sigma, Cat# A9434), NaCl (5 mM; Sigma, Cat# S7653), KCl (5 mM; Sigma, Cat# P5405) or sucrose (10 mM; Sigma, Cat# S7903). Methionine sulfoximine (MSO; 5 mM; Sigma, Cat# M5379) was used to inhibit glutamine synthetase.

### Whole-cell patch-clamp recordings and paired recordings

Whole-cell patch-clamp recordings from CA1 pyramidal neurons of tissue cultures were carried out at 35°C (1–3 cells per culture). For recordings of spontaneous excitatory postsynaptic currents (sEPSCs) the bath solution (artificial cerebrospinal fluid, ACSF) contained 126 mM NaCl, 2.5 mM KCl, 26 mM NaHCO_3_, 1.25 mM NaH_2_PO_4_, 2 mM CaCl_2_, 2 mM MgCl_2_, 10 mM glucose and was saturated with 95% O_2_/5% CO_2_. For recording of intrinsic cellular properties in current-clamp mode, the basic bath solution contained also 10 μM D-APV, 10 μM NBQX (Tocris; Cat# 0373) and 10 μM BMI (Sigma, Cat# 14343) or 10 μM Gabazine (Sigma, Cat# S106). Patch pipettes for sEPSC recordings and for intrinsic cellular properties recordings contained 126 mM K-gluconate, 10 mM HEPES, 4 mM KCl, 4 mM ATP-Mg, 0.3 mM GTP-Na_2_, 10 mM PO-Creatine, and 0.1% (w/v) biocytin (pH 7.25 with KOH, 290 mOsm with sucrose, all from Sigma). Spontaneous excitatory postsynaptic currents (sEPSCs) were recorded at a holding potential of −60 mV for CA1 pyramidal neurons. A holding potential of −80 mV was used for astrocytes in the CA1 subfield of the hippocampus.

Series resistance was monitored before and after each recording, and recordings were discarded if the series resistance reached ≥30 MΩ. In current-clamp mode, I–V curves were generated for CA1 neurons by injecting 1 s square pulse currents, starting at −100 pA and increasing in 10 pA steps up to +40 pA (sweep duration: 2 s). For astrocyte membrane properties, I–V curves were generated by injecting 1 s square pulse currents, starting at −500 pA and increasing in 100 pA steps until +500 pA (sweep duration: 2 s). Resting membrane potential was assessed from the baseline value of the I–V-curve. Input resistance was calculated for the injection of −100 pA (CA1 pyramidal neurons) and − 500 pA (astrocytes) currents, respectively, within a 200 ms time frame at the end of the current step.

For paired whole-cell patch-clamp recordings, action potentials were generated in the presynaptic CA3 pyramidal neuron by square current pulses (1 nA) elicited at 0.1 Hz while recording evoked excitatory postsynaptic currents (eEPSCs) from CA1 pyramidal neurons for 20 min. Wash-in of ACSF containing 5 mM NH_4_Cl started after 10 min of baseline recordings. Neurons were considered connected if >5% of action potentials evoked time-locked inward eEPSCs within 10 ms after action potential induction.

### *Post hoc* identification of recorded neurons and astrocytes

After recording the intrinsic cellular properties, tissue cultures were fixed in a solution of 4% (w/v) paraformaldehyde and 4% (w/v) sucrose in 0.1 M phosphate-buffered saline (PBS) for 1 h at room temperature. After fixation the tissue cultures were washed with 0.1 PBS. Afterwards, the fixed tissue cultures were incubated for 1 h at room temperature in blocking solution consisting of 10% (v/v) normal goat serum (NGS; Fisher Scientific, Cat# NC9270494) and 0.5% (v/v) Triton X-100 in 0.1 M PBS. Biocytin (Sigma-Aldrich, Cat# B4261) filled neurons and astrocytes were stained with Alexa-488 or Alexa-633 conjugated Streptavidin (1:1000 in 0.1 M PBS with 10% NGS and 0.1% Triton X-100; Thermo Fisher Scientific, Cat# S32354 or S21375) overnight at 4°C while shaking. DAPI (Thermo Fisher Scientific, Cat# 62248) or TO-PRO^®^ (Invitrogen, Cat# T-3605) staining was used to visualize cytoarchitecture (1:2000; in 0.1 M PBS for 15 min). Slices were washed three times with 0.1 M PBS, transferred and mounted onto glass slides with anti-fading mounting medium (DAKO; Agilent, Cat# S3023) for visualization. Streptavidin-stained CA1 pyramidal neurons were visualized with a Leica TCS SP8 laser scanning microscope with 20× (NA 0.75; Leica), 40× (NA 1.30; Leica) and 63× (NA 1.40; Leica) oil-submersion objectives.

### Experimental design and statistical analysis

**Figure 1:**
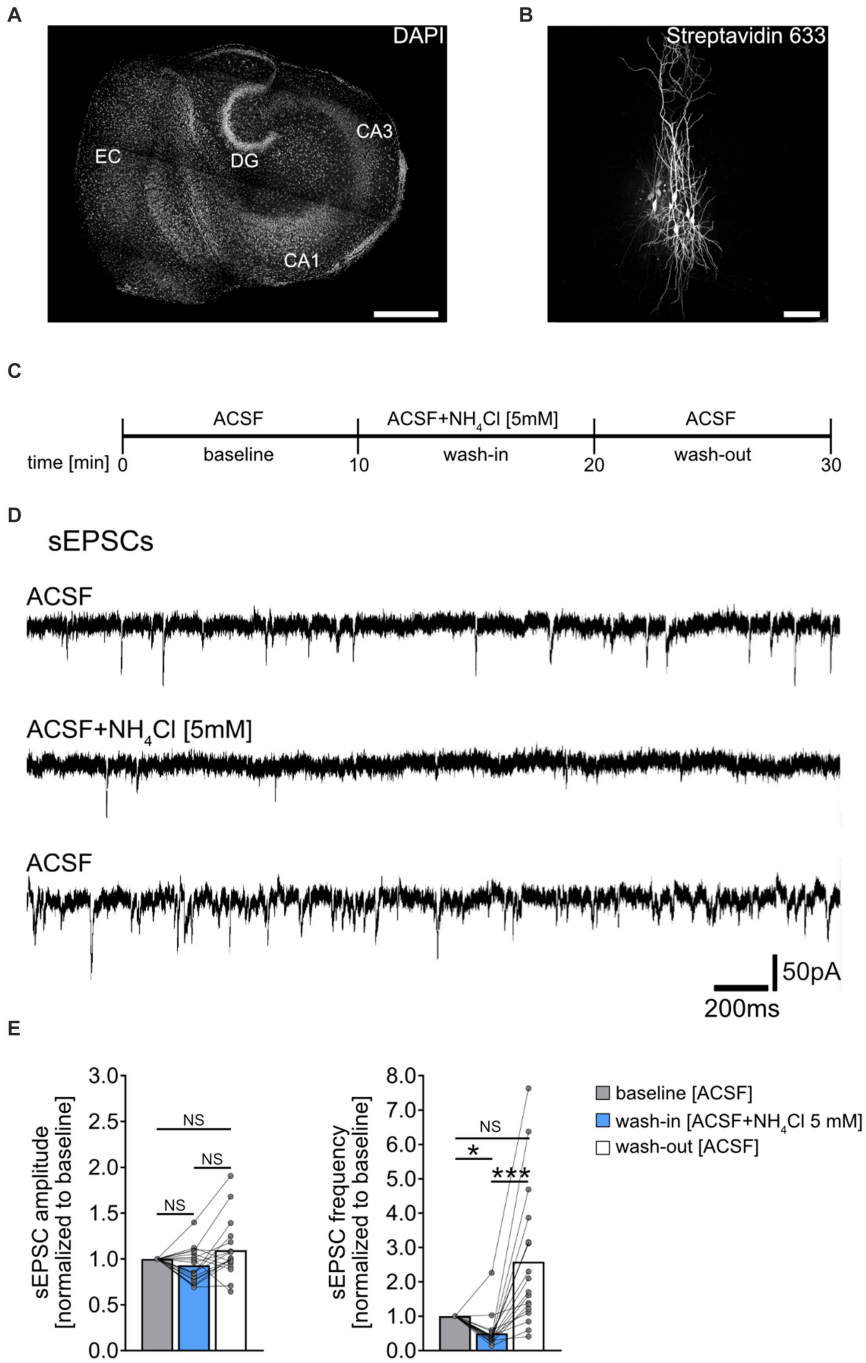
Effect of NH_4_Cl on spontaneous excitatory postsynaptic currents recorded from CA1 pyramidal neurons. **(A)** Overview of a mouse organotypic entorhino-hippocampal tissue culture stained with DAPI (EC, entorhinal cortex; DG, dentate gyrus; CA1, Cornu Ammonis 1; CA3, Cornu Ammonis 3). Scale bar, 400 μm. **(B)** Example of CA1 pyramidal neurons filled with biocytin during recording and *post hoc*-stained with Streptavidin 633. Scale bar, 50 μm. **(C)** Schematic illustration of the experimental design. A 10-min baseline recording was followed by a 10-min wash-in of 5 mM NH_4_Cl, and subsequently, a 10-min wash-out with regular ACSF. **(D,E)** Sample traces and group data of spontaneous excitatory postsynaptic currents (sEPSCs) recorded from CA1 pyramidal neurons before (10 min), during (10 min) and after (10 min) exposure to 5 mM NH_4_Cl (*n* = 18 cells from 11 cultures; 1 cell was excluded from the analysis based on the 3x SD criterion; Friedman test followed by Dunn’s *post hoc* test; for comparison of the mean frequency *p*_baseline-wash-in_ = 0.014; *p*_wash-in-wash-out_ < 0.001). Connected gray dots indicate data points from individual cells, values represent mean ± SEM (***p < 0.001, *p < 0.05; NS, not significant).

**Figure 2:**
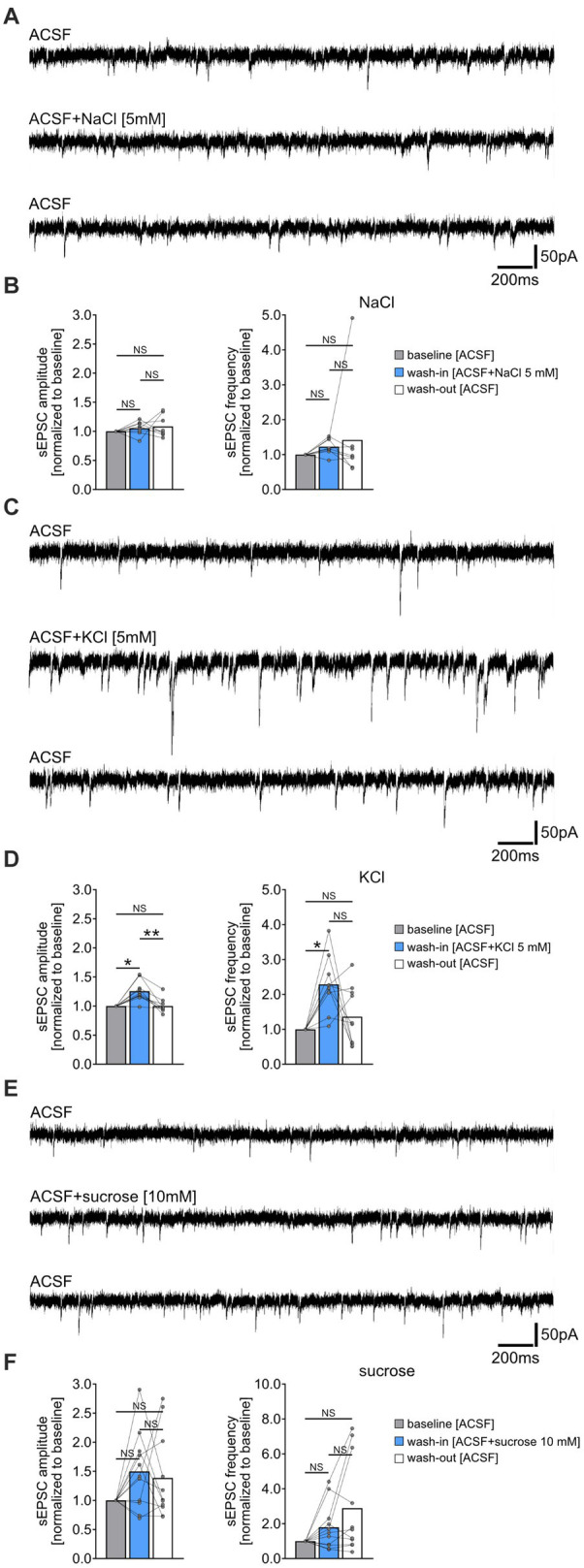
Increased extracellular chloride, potassium or osmolarity do not reduce the frequency of spontaneous excitatory postsynaptic currents (sEPSC). **(A,B)** Sample traces and group data of sEPSCs recorded from CA1 pyramidal neurons of tissue cultures before (10 min), during (10 min), and after (10 min) exposure to 5 mM NaCl (*n* = 8 cells from 8 cultures; Friedman test followed by Dunn’s *post hoc* test). **(C,D)** Sample traces and group data of sEPSCs recorded from CA1 pyramidal neurons of tissue cultures before (10 min), during (10 min), and after (10 min) exposure to 5 mM KCl (*n* = 10 cells from 6 cultures; Friedman test followed by Dunn’s *post hoc* test; for comparison of the mean amplitude: *p*_baseline-wash-in_ = 0.042; *p*_wash-in-wash-out_ = 0.001. For comparison of the mean frequency *p*_baseline-wash-in_ = 0.011). **(E,F)** Sample traces and group data of sEPSCs recorded from CA1 pyramidal neurons of tissue cultures before (10 min), during (10 min), and after (10 min) exposure to 10 mM sucrose (*n* = 11 cells from 4 cultures; Friedman test followed by Dunn’s *post hoc* test). Connected gray dots indicate data points from individual cells, values represent mean ± SEM (**p < 0.01, *p < 0.05; NS, not significant).

Electrophysiological recordings were obtained using a MultiClamp 700B amplifier (Molecular Devices), digitized with Axon Digidata 1550B (Molecular Devices), and analyzed using Clampfit 11 of the pClamp11 software package (Molecular Devices). sEPSC properties were analyzed using the automated template search tool for event detection. Only inward current responses were analyzed in the respective experiments. Clampfit 11 (Molecular Devices^®^) was used to analyze paired recordings and the passive membrane properties of CA1 pyramidal neurons and astrocytes. Statistical comparisons were carried out using GraphPad Prism 7 (GraphPad software). For comparison of two groups Mann–Whitney test was used. In order to statistically compare three groups of paired measurements, Friedman test followed by Dunn’s *post hoc* test was selected. For statistical evaluation of XY-plots, a two-way ANOVA test followed by Sidak’s multiple comparisons test was performed. To compare the mean values of amplitude, failure rate, decay and risetime before and after perfusion of NH_4_Cl for paired recordings, the Wilcoxon matched-pairs signed-rank test was used. In Figure 1E, one cell that had a baseline amplitude, which was higher than three times the standard deviation value compared to the mean was excluded from further analysis (3× SD criterion). In Figure 2B, two cells that had an extremely aberrant increase in their frequency during wash-out (~11- and ~44-fold, respectively) after perfusion with NaCl were excluded from further analysis. In Figure 5, three cells that showed an aberrant increase or decrease in their amplitudes or frequencies under normal non-treated conditions (ACSF-only), with values during the last minute, three times different compared to the values of baseline, were also excluded from further analysis.

### Digital illustrations

Figures were prepared using the Affinity Designer (Serif Europe, Nottingham, United Kingdom) and the Adobe Photoshop (Adobe, San Jose, CA, United States) graphics software. Image brightness and contrast were adjusted.

## Results

### Acute exposure to NH_4_Cl attenuates sEPSC frequency

Spontaneous excitatory postsynaptic currents (sEPSCs) were recorded from individual CA1 pyramidal neurons of entorhino-hippocampal tissue cultures (≥18 days *in vitro;* Figures 1A,B), to assess acute effects of NH_4_Cl on excitatory synaptic activity. Baseline recordings were taken in ACSF for 10 min, followed by a 10-min exposure to 5 mM NH_4_Cl in ACSF, and a 10-min ACSF wash-out (Figures 1C,D). NH_4_Cl significantly reduced sEPSC frequencies without affecting mean sEPSC amplitude (Figure 1E). This effect was not permanent post-wash-out, with the vast majority of cells showing increased sEPSC frequencies afterwards (Figure 1E; baseline vs. post-wash-out; not significantly different statistically). The exact mechanism and reason for the increased sEPSC frequencies after wash-out are currently unknown.

### Increased extracellular chloride, potassium or osmolarity do not reduce the frequency of spontaneous excitatory postsynaptic currents (sEPSCs)

To determine if the NH_4_Cl-mediated reduction in sEPSC frequencies was due to increased extracellular chloride, potassium, or osmolarity, we performed the following control experiments. sEPSCs were recorded from another set of CA1 pyramidal neurons before and after washing-in 5 mM sodium chloride- (NaCl), 5 mM potassium chloride- (KCl) or 10 mM sucrose-containing ACSF (Figure 2). Prior to these experiments, we verified the pH and osmolarity of the solutions, noting an expected increase in osmolarity but not pH changes compared to ACSF alone (Supplementary Figure S1). Acute, i.e., 10 min exposure to 5 mM NaCl containing ACSF did not significantly affect sEPSC amplitudes and frequencies (Figures 2A,B). However, exposure to 5 mM KCl in ACSF led to a reversible enhancement in synaptic activity, marked by increased sEPSC amplitudes and frequencies (Figures 2C,D). The addition of 10 mM sucrose had no significant effect on sEPSC parameters (Figures 2E,F). Notably, we observed an increase in sEPSC frequencies in some cells after washout of NaCl, KCl, and sucrose, suggesting that this effect may not be specific to NH_4_Cl (c.f., Figure 1E). We conclude that the attenuation of sEPSC frequencies by NH_4_Cl is mediated by NH_4_^+^ cations, rather than by changes in extracellular chloride, potassium or osmolarity.

### Synaptic effects of ammonium chloride in CA3/CA1-paired recordings

**Figure 3:**
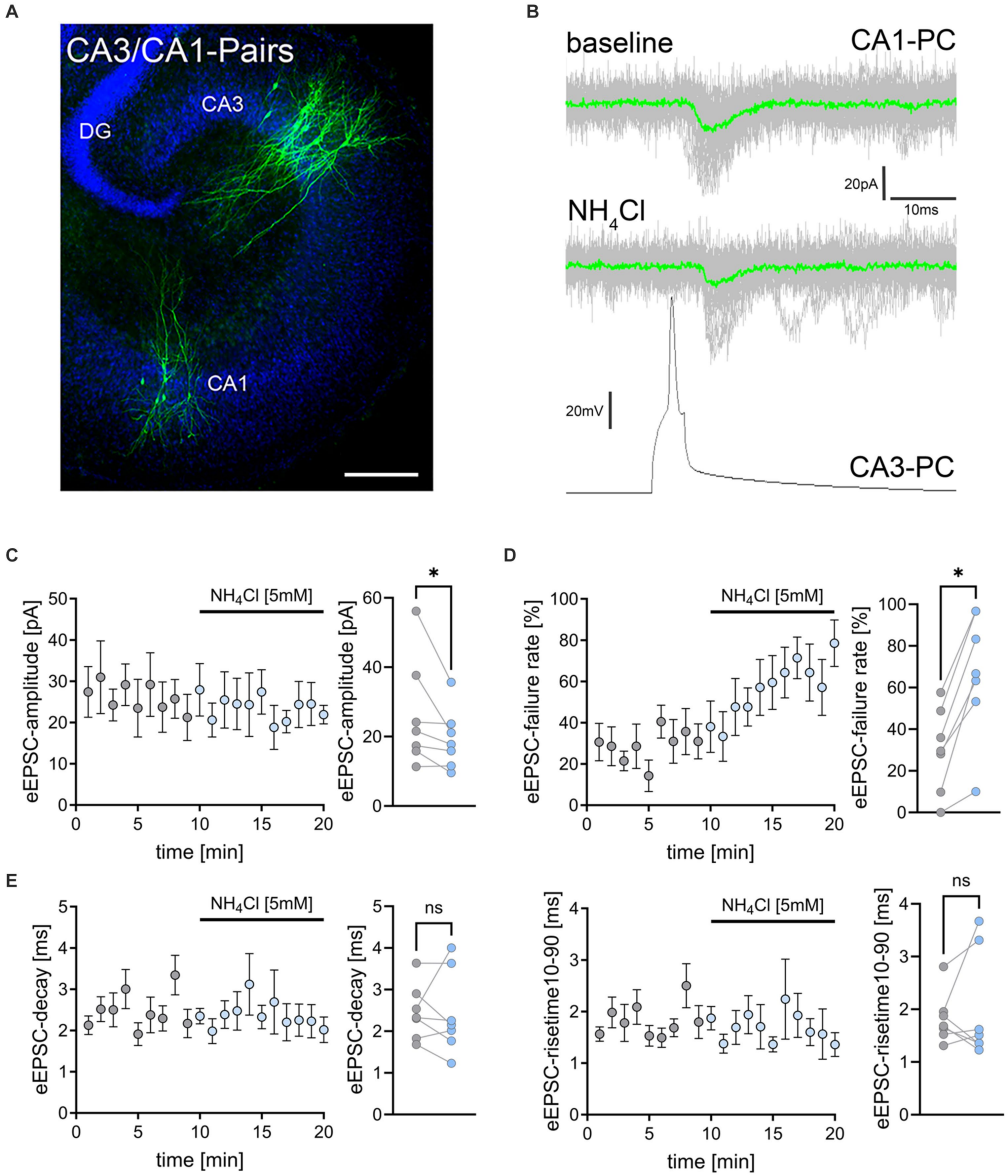
NH_4_Cl reduces the amplitude and increases the failure rate of eEPSCs on CA3-to-CA1 synapses. **(A)** *Post hoc* staining (green) of patched and simultaneously recorded pairs of CA3 and CA1 pyramidal neurons. TO-PRO (blue) was used to visualize cytoarchitecture (DG, dentate gyrus; CA1, Cornu Ammonis 1; CA3, Cornu Ammonis 3) Scale bar, 100 μm. **(B)** Averaged responses of successfully evoked time-locked postsynaptic currents from CA1 neurons. Action potentials were induced at 0.1 Hz in presynaptic CA3 pyramidal neurons while recording evoked excitatory postsynaptic currents (eEPSCs) from CA1 neurons. **(C–E)** Data of amplitude **(C)**, failure rate **(D)**, decay and risetime **(E)** of eEPSCs before (gray) and during (blue) exposure to 5 mM NH_4_Cl [*n* = 7 pairs from 7 cultures; Wilcoxon matched-pairs signed rank test comparing mean values of first 5 min (baseline) and last 5 min (wash-in) in each pair; *p*_amplitude_ = 0.016; *p*_failure rate_ = 0.016]. Values represent mean ± SEM (*p < 0.05; NS, not significant).

To further validate and extend our findings on the effects of NH_4_Cl on excitatory neurotransmission, we conducted simultaneous whole-cell patch clamp recordings on connected pairs of CA3 and CA1 pyramidal neurons (Figure 3A). Action potentials were induced in CA3 neurons at 0.1 Hz, while evoked excitatory postsynaptic currents (eEPSCs) were recorded from CA1 neurons (Figure 3B). Neuronal pairs were considered connected when >5% of presynaptic action potentials triggered time-locked eEPSCs within 10 ms after action potential induction. Following 10-min baseline recordings, 5 mM NH_4_Cl in ACSF was introduced (c.f., Figure 1C). In these experiments we observed a slight but significant reduction in eEPSC amplitudes after wash-in of NH_4_Cl (Figure 3C). Additionally, there was a significant increase in synaptic failure rates, with the percentage of action potentials not evoking postsynaptic responses rising from 24.7 ± 6.51% to 67.1 ± 11.42% (Figure 3D; mean ± SEM, n = 7 pairs from 7 cultures; Wilcoxon matched-pairs signed-rank test comparing mean values of first 5 min (baseline) and last 5 min (wash-in) in each pair; *p* = 0.016). However, eEPSC decay times and rise times were not significantly affected (Figure 3E). These findings confirmed that NH_4_Cl impacts excitatory synaptic transmission.

### NH_4_Cl alters the passive membrane properties of astrocytes while leaving membrane properties of CA1 neurons unaffected

**Figure 4:**
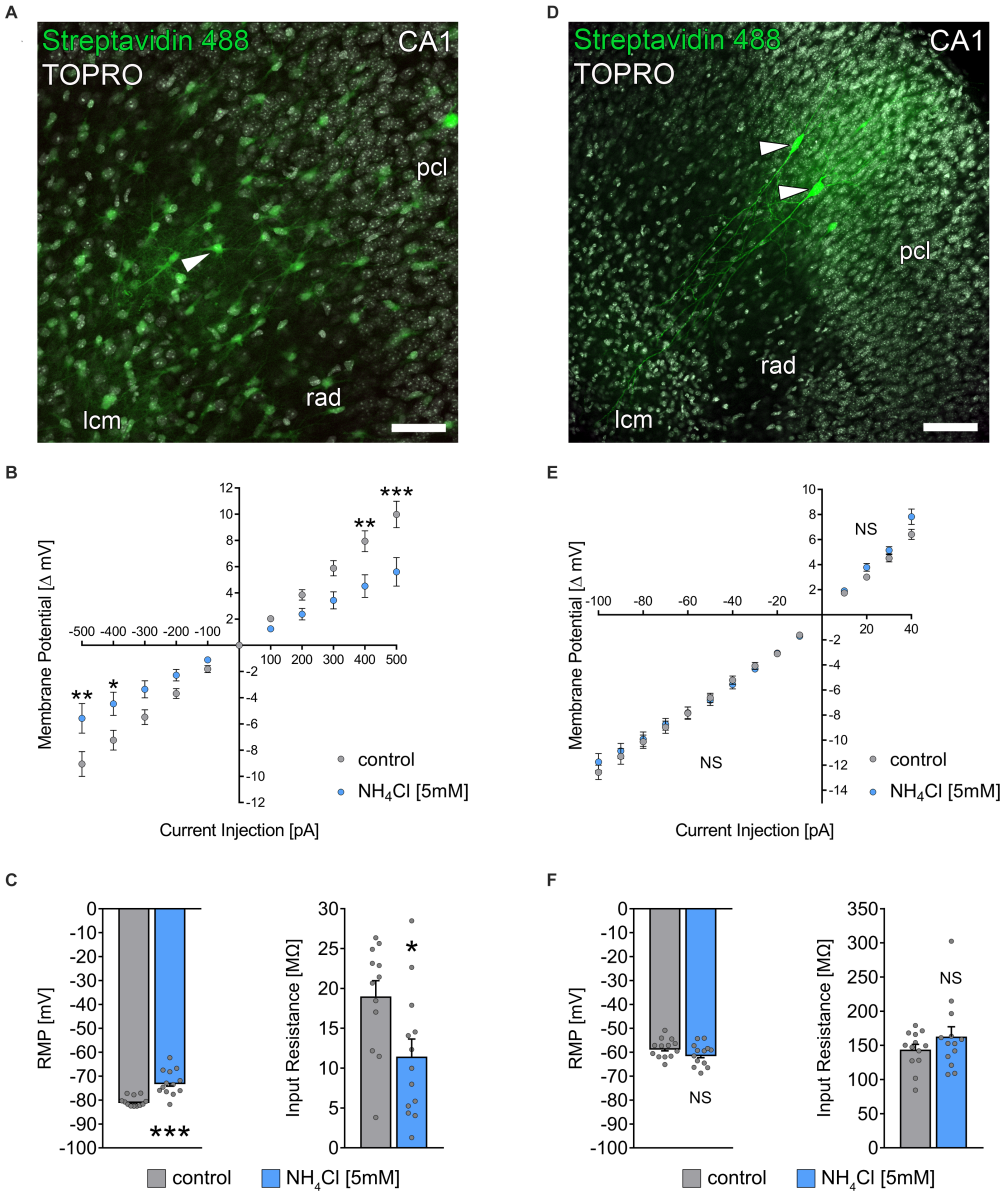
NH_4_Cl alters the passive membrane properties of astrocytes. **(A)** *Post hoc* staining of recorded biocytin-filled astrocytes (arrowhead) in the stratum radiatum of CA1. TO-PRO (gray) was used to visualize cytoarchitecture (pcl, stratum pyramidale; rad, stratum radiatum; lcm, stratum lacunosum-moleculare). Scale bar, 50 μm. **(B)** Group data of input–output curves of astrocytes in non-treated and 5 mM NH_4_Cl-treated cultures (*n*_control_ = 12 cells from 3 cultures; *n*_NH4Cl_ = 13 cells from 4 cultures; 2way ANOVA followed by Sidak’s multiple comparisons test; *p*_−500_ = 0.003; *p*_−400_ = 0.037; *p*_400_ = 0.004; *p*_500_ < 0,001). **(C)** Group data of resting membrane potentials and input resistances of astrocytes in non-treated and 5 mM NH_4_Cl-treated cultures (*n*_control_ = 12 cells from 3 cultures; *n*_NH4Cl_ = 13 cells from 4 cultures; Mann–Whitney test; *p*_RMP_ < 0,001; *p*_Input Resistance_ = 0.03). **(D)** *Post hoc* staining of recorded biocytin-filled CA1 pyramidal neurons (arrowheads). Scale bar, 100 μm. **(E)** Group data of input–output curves of CA1 pyramidal neurons in non-treated and 5 mM NH_4_Cl-treated cultures (*n*_control_ = 13 cells from 4 cultures; *n*_NH4Cl_ = 13 cells from 4 cultures; 2way ANOVA followed by Sidak’s multiple comparisons test). **(F)** Group data of resting membrane potentials and input resistances of CA1 pyramidal neurons in non-treated and 5 mM NH_4_Cl-treated cultures (*n*_control_ = 13 cells from 4 cultures; *n*_NH4Cl_ = 13 cells from 4 cultures; Mann–Whitney test). Gray dots indicate individual data points, values represent mean ± SEM (***p < 0.001, **p < 0.01, *p < 0.05; NS, not significant).

Previous research has shown that ammonia can cause astrocytic swelling (Jayakumar et al., 2009) and disrupt astrocytic glutamate uptake (Rose, 2006). Because we observed a reduction in excitatory neurotransmission, we wondered whether an effect on astrocytes was also present in our experimental setting. To investigate if NH_4_Cl affects astrocytic membrane properties, we patched individual astrocytes in stratum radiatum of the hippocampal CA1 region in control cultures exposed to ACSF-only and cultures exposed to ACSF containing 5 mM NH_4_Cl (for 10 min) immediately before recordings (Figures 4A–C). Interestingly, NH_4_Cl exposure resulted in an increased resting membrane potential (RMP) and decreased input resistance in astrocytes (Figures 4B,C). To determine the specificity of NH_4_Cl effect on astrocytes, we also recorded passive membrane properties of CA1 pyramidal neurons in ACSF-only or ACSF with 5 mM NH_4_Cl (different sets of cultures; 10 min; Figures 4D–F). These recordings showed no significant changes in RMP and input resistance between the two groups (Figures 4E,F), suggesting that at 5 mM NH_4_Cl specifically alters the passive membrane properties of astrocytes without significantly affecting those of CA1 neurons.

### Inhibition of glutamine synthetase prevents the NH_4_Cl-mediated weakening of sEPSC frequency

**Figure 5:**
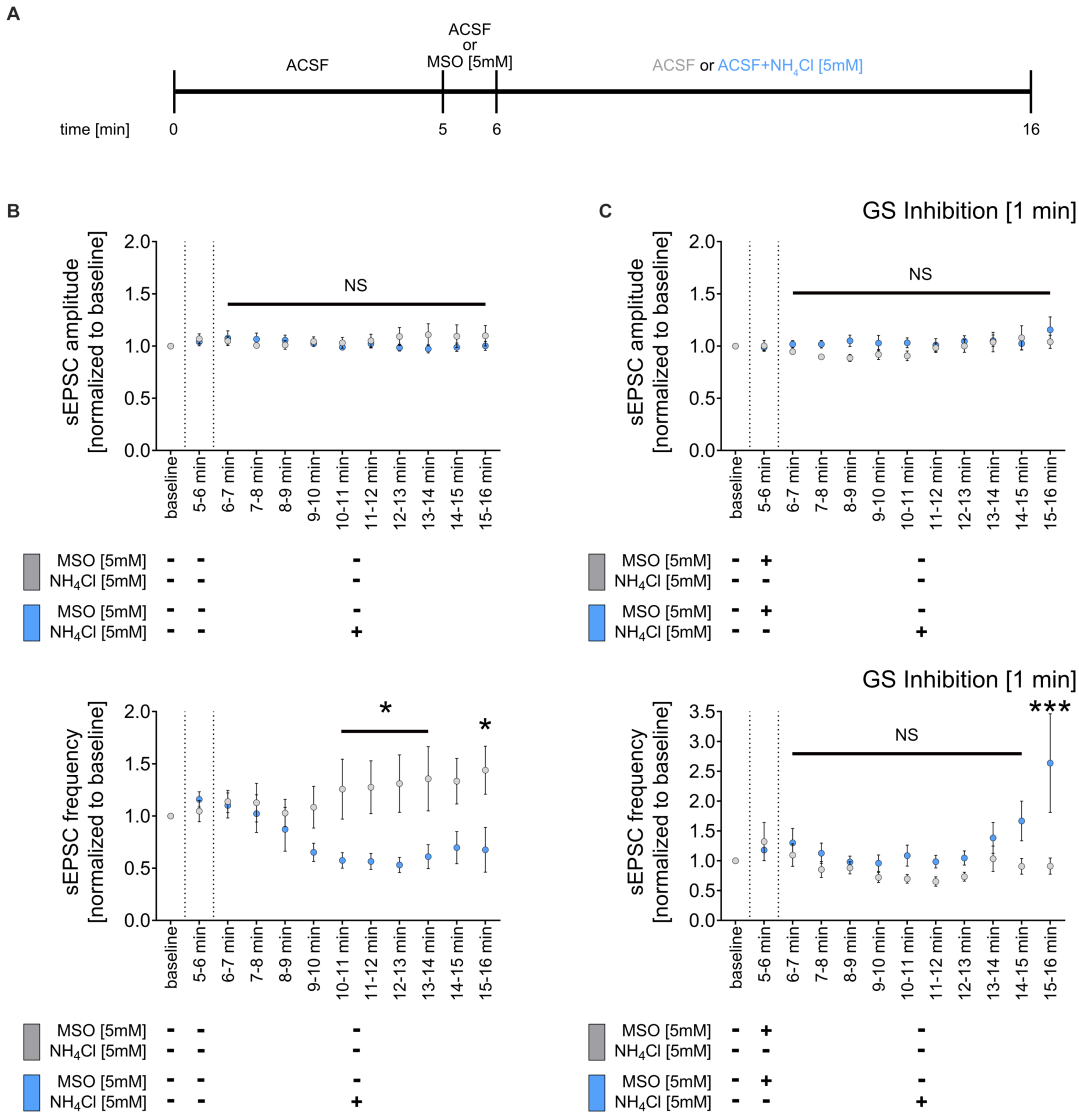
Inhibition of glutamine synthetase prevents the NH_4_Cl-induced reduction of sEPCS frequency. **(A)** Schematic illustration of the experimental design. Baseline was recorded for 5 min, followed by 1-min wash-in of 5 mM MSO or normal ACSF and a subsequent 10-min wash-in of 5 mM NH_4_Cl or normal ACSF. **(B)** Group data of spontaneous excitatory postsynaptic currents (sEPSCs) recorded from CA1 pyramidal neurons after wash-in of 5 mM NH_4_Cl or normal ACSF (control) for 10 min (*n*_control_ = 9 cells from 6 cultures; *n*_NH4Cl_ = 16 cells from 10 cultures; 2way ANOVA followed by Sidak’s multiple comparisons test; *p*_10-11 min_ = 0.049; *p*_11-12 min_ = 0.034; *p*_12-13 min_ = 0.013; *p*_13-14 min_ = 0.021; *p*_15-16 min_ = 0.017). **(C)** Group data of spontaneous excitatory postsynaptic currents (sEPSCs) recorded from CA1 pyramidal neurons of tissue cultures exposed to 5 mM MSO for 1 min, followed by 10-min wash-in of 5 mM NH_4_Cl or normal ACSF (control) (*n*_control + MSO_ = 10 cells from 6 cultures; *n*_NH4Cl + MSO_ = 12 cells from 7 cultures; 2way ANOVA followed by Sidak’s multiple comparisons test; *p*_15-16 min_ < 0.001). Values represent mean ± SEM (***p < 0.001, *p < 0.05, NS, not significant).

Glutamine synthetase, primarily expressed by astrocytes in the central nervous system (Derouiche and Frotscher, 1991), is essential for ammonia and synaptic glutamate clearance (Suarez et al., 2002). We theorized that NH_4_Cl exposure might reduce sEPSC frequencies via astrocytic glutamine synthetase, which converts glutamate to glutamine when NH_4_Cl is present. This process potentially diminishes the availability of glutamate, impacting excitatory synapses. To test this, we initially recorded a 5-min sEPSC baseline, followed by a 1-min wash-in of ACSF containing 5 mM methionine sulfoximine (MSO) (Figure 5A), an irreversible inhibitor of glutamine synthetase (Jeitner and Cooper, 2014). Subsequently, we introduced either NH_4_Cl-containing or regular ACSF for 10 min (Figure 5A). As before, 5 mM NH_4_Cl reduced the mean sEPSC frequency without altering sEPSC amplitudes (Figure 5B). However, pre-treatment with 5 mM MSO did not affect sEPSC amplitudes and frequencies but did prevent the NH_4_Cl-induced decrease in sEPSC frequencies, even reversing it after 10 min (Figure 5C). The exact mechanism and reason for the increased sEPSC frequencies are currently unknown. Overall, these results suggest that the reduction of sEPSC frequencies is mediated through glutamine synthetase. This underscores the role of astrocytes in synaptic changes induced by NH_4_Cl.

## Discussion

This study demonstrated that acute NH_4_Cl exposure reduces excitatory synaptic transmission in CA1 pyramidal neurons of organotypic slice cultures. While NH_4_Cl did not significantly alter the passive membrane properties of CA1 pyramidal neurons, it notably affected astrocytes by increasing their RMP and changing their membrane input resistance. The prevention of NH_4_Cl-induced reduction in excitatory synaptic activity by inhibition of glutamine synthetase suggests that this enzyme in astrocytes primarily mediates the detrimental effect of NH_4_Cl on excitatory synaptic transmission.

Prior research has examined the effects of NH_4_Cl on synaptic plasticity [reviewed in Wen et al. (2013)], with a focus on how sustained hyperammonemia affects activity-dependent synaptic plasticity such as long-term potentiation (LTP) and long-term depression (LTD) of excitatory neurotransmission (e.g., Citri and Malenka, 2008). For example, hyperammonemic states have demonstrated detrimental effects on tetanus-induced LTP in hippocampal slices obtained from hyperammonemic rats (Chepkova et al., 2017; Monfort et al., 2007; Munoz et al., 2000) and in cortico-striatal slices in a model of mild HE, specifically, portocaval anastomosis (Sergeeva et al., 2005). Acute NH_4_Cl exposure has disrupted LTP in acute hippocampal slices (Chepkova et al., 2006) and impaired LTD in the cortico-striatal pathway (Chepkova et al., 2012). Our experiments align with these findings, showing acute NH_4_Cl exposure leads to a reduction in sEPSC frequencies, increased synaptic failure rate and reduced amplitudes in connected CA3-CA1 neuron pairs (Lazarenko et al., 2017). The implications of our findings extend to the understanding of NH_4_Cl effects on synaptic plasticity: NH_4_Cl may alter neuronal activity responses to stimulation, which in turn could affect LTP and LTD induction. This does not necessarily reflect intrinsic changes in the neurons’ ability to express synaptic plasticity or alterations in LTP/LTD *per se*.

Another important aspect to consider is whether the chronic effects of NH_4_Cl are directly attributed to ammonia or if they partly reflect compensatory homeostatic mechanisms counteracting synaptic effects of NH_4_Cl. Indeed, chronic hyperammonemia has been linked to modifications of AMPA receptor composition, possibly indicating a postsynaptic compensatory response. Specifically, long-term ammonia exposure leads to the increased insertion of GluA2-containing, Ca^2+^-impermeable AMPA receptors into the membrane (Taoro-Gonzalez et al., 2018; Taoro-Gonzalez et al., 2019), influencing the ability of neurons to express plasticity crucial for learning and memory (Hollmann et al., 1991; Wiltgen et al., 2010). Our previous work suggested that activated microglia may mediate synaptic homeostasis, (Kleidonas et al., 2023), potentially affecting plasticity-induction under specific pathological conditions [for review: (Cornell et al., 2022; You et al., 2024)]. Moreover, microglia activation is associated with increased inhibition and synaptic plasticity alterations (Abareshi et al., 2016; Izumi et al., 2021; Jiang et al., 2022; Lenz et al., 2020; Strehl et al., 2014), as observed in chronic HE (Sancho-Alonso et al., 2022a). It is evident that further investigation is required to clarify the interplay between NH_4_Cl exposure, changes in excitatory neurotransmission, microglia activation, and maladaptive homeostatic synaptic plasticity.

Alterations in the structure and function of astrocytes play a significant role in HE pathology (Claeys et al., 2021; Elsherbini et al., 2022; Jaeger et al., 2019; Lu et al., 2019). Traditionally, astrocytic swelling was attributed to ammonia exposure, but Rangroo Thrane et al. (2013) presented evidence challenging this view, suggesting that impaired potassium buffering by astrocytes underlies acute *in vivo* effects of ammonia. Compelling evidence underscores that ammonia prompts oxidative and nitrosative stress, specifically altering astrocytic but not neuronal functions (Gorg et al., 2019; Gorg et al., 2013; Haussinger et al., 2022; Lachmann et al., 2013). Our findings corroborate the vulnerability of astrocytes to NH_4_Cl; we observed that acute NH_4_Cl exposure results in hippocampal astrocytic depolarization and a decrease in their input resistance (c.f., Stephan et al., 2012). Notably, these alterations do not extend to the passive membrane properties of CA1 pyramidal neurons, contrasting observations in acute hippocampal slices (Kelly and Church, 2005). This may suggest that the neuronal effects of NH_4_Cl may vary between acutely prepared brain slices and organotypic tissue cultures, which are allowed an 18-day post-preparation recovery period. Thus, investigating the impact of NH_4_Cl on acutely lesioned networks *in vitro*, *and in vivo* could further elucidate the complex pathology of hyperammonemia, offering new insights into its effects on brain tissue. Regardless of these considerations, it remains to be shown whether the effects of NH_4_Cl on astrocytes and excitatory synaptic transmission reported in this study can be replicated in the hippocampus and other brain regions of the intact brain.

Despite these considerations, our research demonstrated that pharmacological inhibition of glutamine synthetase mitigates the NH_4_Cl-induced reduction in sEPSC frequencies. This finding implies a significant recruitment of astrocytic glutamine synthetase early in NH_4_Cl exposure. Given that astrocytic glutamine synthetase plays a crucial role in converting ammonium and glutamate into glutamine (Rose et al., 2013), its activation supports the swift removal of glutamate from the synaptic cleft by astrocytes (Magi et al., 2019), which may affect synaptic activity. Excitatory amino acid transporters 1 and 2 (EAAT1 and EAAT2) on astrocytic plasma membranes might be involved in this process (Todd and Hardingham, 2020). However, further research is required to unravel the complex mechanisms and consequences of these findings, including the observed shifts in astrocyte RMP and input resistance. Determining whether the early heightened activity of glutamine synthetase, the suppression of excitatory transmission, and alterations in astrocyte RMP and input resistance culminate in compensatory maladaptive adjustments, and NH_4_Cl-induced dysfunctions during prolonged hyperammonemia remains a critical inquiry. We are confident that taking into account these temporal dynamics, along with the possible involvement of (microglia-mediated maladaptive) homeostatic plasticity, will aid in understanding the pathomechanisms of HE.

## Data Availability

The original contributions presented in the study are included in the article/Supplementary material, further inquiries can be directed to the corresponding author.

## Supplementary material

SUPPLEMENTARY FIGURE S1 Osmolarity, but not the pH, of ACSF is affected by 5 mM NH_4_Cl. **(A)** Group data of pH values of: ACSF, ACSF with 5 mM NH_4_Cl, ACSF with 5 mM KCl, ACSF with 5 mM NaCl and ACSF with 10 mM sucrose (*n* = 5 samples per group; Ordinary one-way ANOVA followed by Tukey’s multiple comparisons test). **(B)** Group data of osmolarity values of: ACSF, ACSF with 5 mM NH_4_Cl, ACSF with 5 mM KCl, ACSF with 5 mM NaCl and ACSF with 10 mM sucrose (*n* = 5 samples per group; Ordinary one-way ANOVA followed by Holm–Sidak’s multiple comparisons test; pNH_4_Cl = 0,009; *p*_KCl_ <0,001; *p*_NaCl_ <0,001; *p*_sucrose_ <0,001). Values represent mean ± S.E.M. (***p*<0.001, **p*<0.05; NS, not significant).
