# Supplementary figures and images for: Ammonium chloride reduces excitatory synaptic transmission onto CA1 pyramidal neurons of mouse organotypic slice cultures
Source: Front Cell Neurosci. 2024 Oct 1;18:1410275. doi: 10.3389/fncel.2024.1410275 (PMC11473415; doi:10.3389/fncel.2024.1410275)

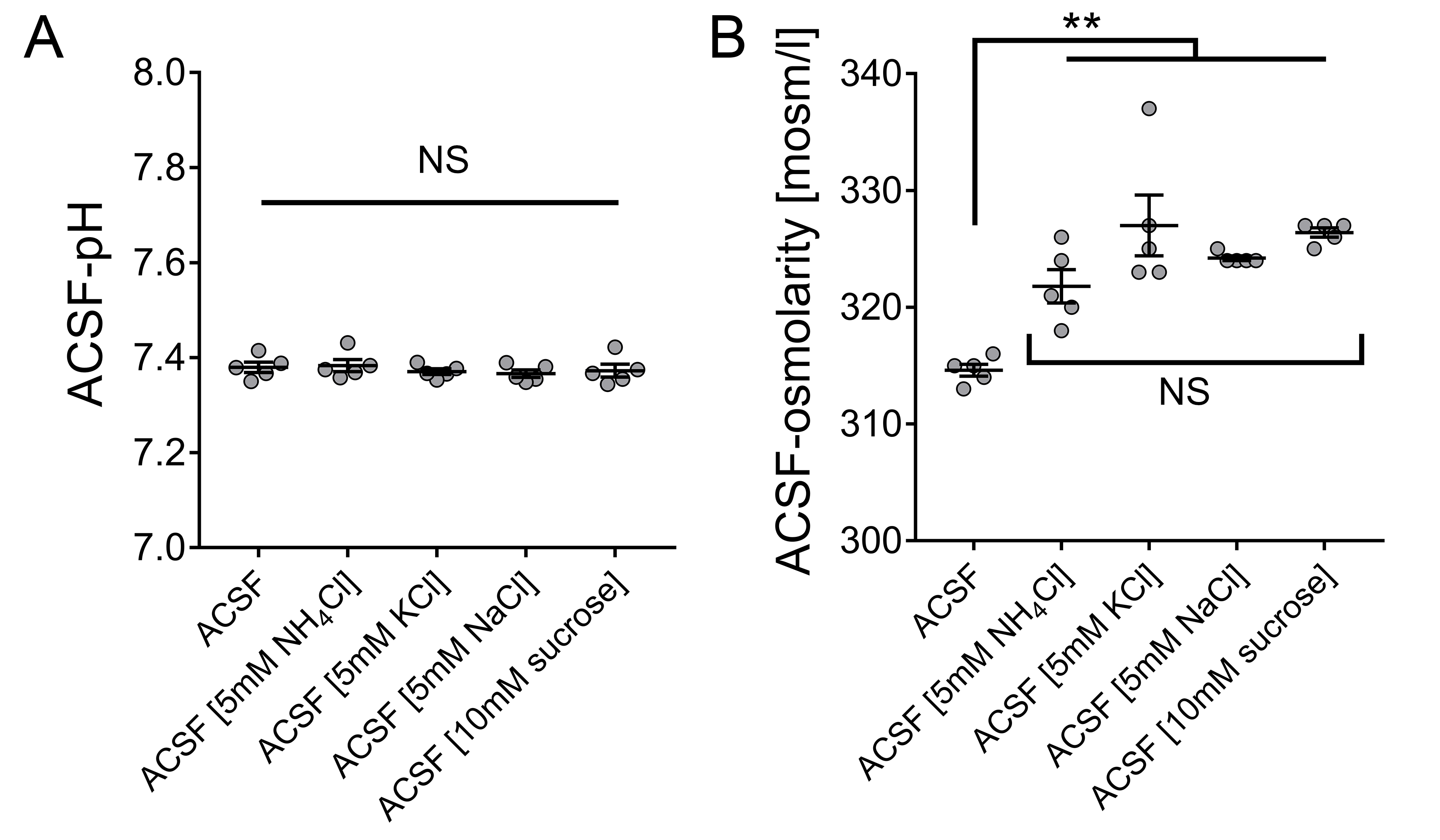

Supplement: SUPPLEMENTARY FIGURE S1 — Osmolarity, but not the pH, of ACSF is affected by 5 mM NH4Cl. (A) Group data of pH values of: ACSF, ACSF with 5 mM NH4Cl, ACSF with 5 mM KCl, ACSF with 5 mM NaCl and ACSF with 10 mM sucrose (n = 5 samples per group; Ordinary one-way ANOVA followed by Tukey’s multiple comparisons test). (B) Group data of osmolarity values of: ACSF, ACSF with 5 mM NH4Cl, ACSF with 5 mM KCl, ACSF with 5 mM NaCl and ACSF with 10 mM sucrose (n = 5 samples per group; Ordinary one-way ANOVA followed by Holm–Sidak’s multiple comparisons test; pNH4Cl = 0,009; pKCl <0,001; pNaCl <0,001; psucrose <0,001). Values represent mean ± S.E.M. (**p<0.001, *p<0.05; NS, not significant). [file Image_1.TIFF]
